# Host–Microbiota Mutualism in Metabolic Diseases

**DOI:** 10.3389/fendo.2017.00267

**Published:** 2017-10-04

**Authors:** Salvatore Fabbiano, Nicolas Suárez-Zamorano, Mirko Trajkovski

**Affiliations:** ^1^Faculty of Medicine, Department of Cell Physiology and Metabolism, University of Geneva, Centre Médical Universitaire (CMU), Geneva, Switzerland; ^2^Faculty of Medicine, University of Geneva, Diabetes Center, Geneva, Switzerland; ^3^Institute for Genetics and Genomics of Geneva (iGE3), University of Geneva, Geneva, Switzerland

**Keywords:** microbiota, obesity, metabolism, dysbiosis, diet, probiotics, fecal transplant, co-metabolism

## Abstract

The intestinal microbiota is a plastic ecosystem that is shaped by environmental and genetic factors, interacting with virtually all tissues of the host. Many signals result from the interplay between the microbiota with its mammalian symbiont that can lead to altered metabolism. Disruptions in the microbial composition are associated with a number of comorbidities linked to the metabolic syndrome. Promoting the niche expansion of beneficial bacteria through diet and supplements can improve metabolic disorders. Reintroducing bacteria through probiotic treatment or fecal transplant is a strategy under active investigation for multiple pathological conditions. Here, we review the recent knowledge of microbiota’s contribution to host pathology, the modulation of the microbiota by dietary habits, and the potential therapeutic benefits of reshaping the gut bacterial landscape in context of metabolic disorders such as obesity.

## Introduction

The intestinal microbiota is a highly dynamic ecosystem in which hundreds of bacterial species and other microorganisms coexist along with their neighboring mammalian cells. Estimated numbers vary across studies, but it is believed that there are at least as many bacteria as host cells in humans, if not drastically more (1). The most abundant phyla in humans and rodent models are *Proteobacteria, Firmicutes, Actinobacteria*, and *Bacteroidetes*, sharing functional structure among hosts species despite having low taxonomic identity (2). Environmental factors such as early microbial exposure and lifestyle, as well as host genetics, shape its composition and function (3, 4). The gut microbiota in turn affects the host metabolic phenotype, contributes to food and drug metabolism, and helps the immune system to develop (5). From the first observation that obese individuals have a distinct gut microflora compared to lean people (6), and the following efforts to elucidate the function of this altered microbiota (7), the past 10 years have seen a growing body of evidence on the impact of the gut microflora on the host. By transplanting the gut microbiota to germ-free (GF) animals, it has become possible to directly assess the causality of microbiota composition with diseases. In this review, we focus on the relationship between gut microbiota composition and host pathophysiology, and on how shaping the microbiota can be beneficial to promote host health and combat metabolic disorders.

## Deregulation of the Gut Microbiota and the Metabolic Syndrome

### Microbiota Compositional Changes in Metabolic Disorders

The gut microbiota is sensitive to external cues that can reshape it to new stable compositions, resulting sometimes in a deranged, or dysbiotic gut flora. Dysbiotic states are often associated with metabolic alterations in both humans and rodent models such as obesity (6), type 2 diabetes (8), non-alcoholic fatty liver disease (NAFLD) spectrum (9), and dyslipidemia (10). These metabolic traits are often clustered in metabolic syndrome patients (11); it is, therefore, relevant, but challenging, to distinguish the implication of the gut microbiota with each of these pathologies separately.

Germ-free mice are extensively used as a model for studying the importance of the dysbiotic gut flora. Interestingly, GF mice are resistant to obesity following high-fat diets (HFD), and their colonization leads to an increased adiposity along with decreased insulin sensitivity (12) and altered lipid metabolism (13). Nevertheless, when colonization occurs from an obese dysbiotic donor, recipient mice gain even more adiposity and increase their systemic inflammation (14). Considering that HFD challenges elicit heterogeneous responses in terms of weight gain and glucose homeostasis, the differences in the pre-HFD gut microbiota–host interactions in mice can be predictive of the diet outcome (15), where final HFD-driven microbial differences are determinant to transfer these acquired phenotypes following microbiota transplantation and diet challenge (16). Specifically, Le Roy et al. show that GF mice populated with microbiota from two donors similarly obese but discordant in glycemia phenocopy their response to HFD, with a similar increase in the body weight but hyperglycemia and steatosis only in one group. This suggests that components of the gut microbiota can influence liver steatosis and hyperglycemia independently from their effect on adiposity and systemic inflammation.

Dysbiotic microbial composition can be, at least in the case of the obesogenic microbiota, resilient over time. Whereas dieting rapidly reverses the metabolic defects associated with HFD, the dysbiosis provoked in mice after a 4-week HFD persists up to 21 weeks after returning to normal chow diet (17). Importantly, this persistent post-HFD dysbiotic microbiota is not sufficient to drive obesity by itself, but can induce weight gain and glucose intolerance upon exposure to a second HFD stimulus. This two-step obesogenic mechanism relies on a reduced bioavailability of flavonoids (dietary compounds that can promote brown adipose tissue activation and increase energy expenditure) due to the combination of their scarcity in high-fat food and increased flavonoid-degrading ability of the obese microbiota (17). Weight gain upon second exposure to calorie-rich food is a common problem in dieting individuals. Human data are, therefore, needed to assess the plasticity of the microbiota in obese individuals and determine an ideal diet length and composition that would be accompanied with complete and lasting microbial reshaping (17). Another evidence that dysbiosis by itself may not be sufficient to drive metabolic defects comes from the observation that transplant of dysbiotic microbiota to healthy conventional mice neither causes metabolic dysfunctions nor alters the hepatic metabolism (18).

Microbiome analysis on two independent human cohorts described an intestinal microbial signature predicting the glycemic status (19, 20). The stratification of the microbiota analysis for metformin medication highlighted a commonly deregulated pathway in untreated type-2 diabetes (T2D) patients, characterized by decreased abundance of bacteria such as *Roseburia* spp. and *Subdoligranulum* spp., which produce butyrate, a known regulator of hepatic function through intestinal gluconeogenesis (8). Indeed, the metformin in part improves T2D by rescuing the decreased butyrate production through reshaping the microbiota, since microbial transplant from metformin-treated patients was sufficient to improve glucose control in GF mice (21, 22). Of note, other studies using meta-analyses, however, called for the need of large human cohorts to further generalize the predictive power of the microbiota (23–25).

### Microbiota-Driven Regulation of Metabolism

Absence of microbiota in GF mice or through antibiotic treatment improves glucose and lipid metabolism (12, 13, 26, 27), protecting against diet-induced metabolic diseases. These improvements can, at least in part, be explained by increased activity of the thermogenic fat depots (26, 27), and can be reversed by microbial recolonization of the microbiota-depleted animals (27). Cold exposure, the most potent environmental trigger for brown and beige fat development and activation (28), drastically reshapes microbiota composition. Transplantation of this cold-adapted microbiota to GF mice is sufficient to induce tolerance to cold, improve insulin sensitivity, increase energy expenditure, and lower their fat content, largely due to increased brown and beige fat activity in the cold-microbiota transplanted mice (26, 29).

The complexity of the gut microbiota is reflected in its interplay with the host, with a great variety of signaling cues and relay organs (summarized in Table 1). Bile acids (BAs) are released after a meal directly in the proximal intestinal lumen and help lipid absorption by enterocytes. Since around 95% of BAs are reabsorbed in the distal intestine, the total BA pool is relatively stable across the enterohepatic circulation. The gut microbiota metabolizes primary BAs produced by the liver giving rise to secondary BAs, and this microbiota–liver cross talk is responsible of the BA pool (30). BAs act as signaling molecules through intracellular farnesoid X receptor with effect on the overall metabolism (31–35) and membrane-bound G-coupled bile acid receptor (TGR5). TGR5 stimulates intestinal glucagon-like peptide 1 (GLP1) production, brown fat activity, and improves hepatic metabolism in obese animals (36, 37). Interestingly, BAs signaling on intestinal cells can trigger their antimicrobial action (38), suggesting a negative feedback loop. In addition, it was suggested that the brown adipose tissue can also intervene into the gut microbiota–liver regulation of BA pool, since changes in cholesterol metabolism due to the brown adipose tissue activity during cold exposure can increase BAs biosynthesis and drive compositional changes in the gut microbiota (39).

**Table 1:** List of microbe-derived signals that can impact host metabolism.

Signal	Target organ	Effect	Reference
Bile acids (BAs)	Adipose tissue, intestine, liver	Hepatic metabolism, bacterial regulation, lipid metabolism	(36, 38)
Short chain fatty acids (SCFAs)	Adipose tissue, brain, intestine, liver, muscle	Lipid metabolism, regulation of appetite	(40–42)
Neuroactive molecules [g-aminobutyric acid (GABA), serotonine]	Central and peripheral nervous system	Regulation of appetite	(43, 44)
Lipopolysaccharide (LPS)	Adipose tissue, liver, brain	Systemic inflammation, hepatic glucose metabolism, adipose tissue fibrosis	(45–47)
Trimethylamine *N*-oxide	Adipose tissue, liver, kidney	Higher atherosclerosis risk, reduced beige fat	(48, 49)
Branched-chain amino acids (BCAAs)	Adipose tissue, endothelium, skeletal muscle	Adipogenesis, lipid trafficking, lipogenesis, and insulin resistance	(12, 13, 26, 27, 50, 51)

*LPS, BAs, SCFAs, BCAAs, trimethylamine N-oxide, and neuroactive molecules are major known signals of microbial origin that can affect different metabolic organs listed together with the proposed model of action*.

Short chain fatty acids (SCFAs) derive from bacterial fermentation of dietary fibers. They can enter circulation and signal through their cognate receptors to many organs (52, 53) including the central nervous system, which in turn regulates other tissues (40). The SCFA acetate can act in the gut–brain communication, by directly suppressing appetite through hypothalamic activation (41). Conversely, evidence suggested that increased acetate levels in HFD microbiota relay into the parasympathetic nervous system activation driving ghrelin secretion and glucose-stimulated insulin secretion, leading to hyperphagia and metabolic syndrome (42). Other SCFAs are also involved in energy regulation through the gut–brain axis after being sensed in the portal vein and signaling to the autonomous nervous system (54). The gut microbiota also produces or controls the synthesis of other neuroactive signals that can affect the enteric and central nervous system, like g-aminobutyric acid (43) and serotonin (44), both of which could influence appetite and energy balance (55, 56). The contribution of the microbiota-produced neuropeptides to these mechanisms is under active investigation (57).

A group of receptors that senses bacteria-derived metabolites and has been implicated in metabolism is the toll-like receptor family, with TLR2 and TLR4 being particularly important (58). Lipopolysacharide (LPS), a component of the bacterial wall of Gram-negative species, plays a major role in metabolism pathophysiology. Metabolic endotoxemia, in part caused by increased LPS production, is a common consequence of high caloric diets and can affect host metabolism by inducing systemic inflammation and adipose tissue fibrosis, as well as decreasing thermogenesis and hepatic glucose metabolism (45–47). Accordingly, genetic inactivation of TLR4 in hematopoietic cells protects from NAFLD occurrence in mice housed at thermoneutrality (59).

An example of microbial–host interaction is trimethylamine *N*-oxide (TMAO), a product of the co-metabolism of commensal bacteria, producing trimethylamine from dietary precursors, and the liver, which metabolizes it into TMAO through the flavin monooxigenase proteins family (FMOs). Thus, TMAO levels depend on diet, commensal bacteria, and genetics of the host (48). Plasma levels of TMAO have been associated to atherosclerotic plaques and stroke risk in the past (60). Surprisingly, while the knock-out of *Fmo3*, the main enzyme for TMAO production, protects against HFD in mice by promoting beige fat development (49), chronic TMAO infusion improves glucose control and increases insulin secretion *in vivo* and *in vitro* by reducing endoplasmic reticulum stress potentially through a chaperon property (15).

**Figure 1:**
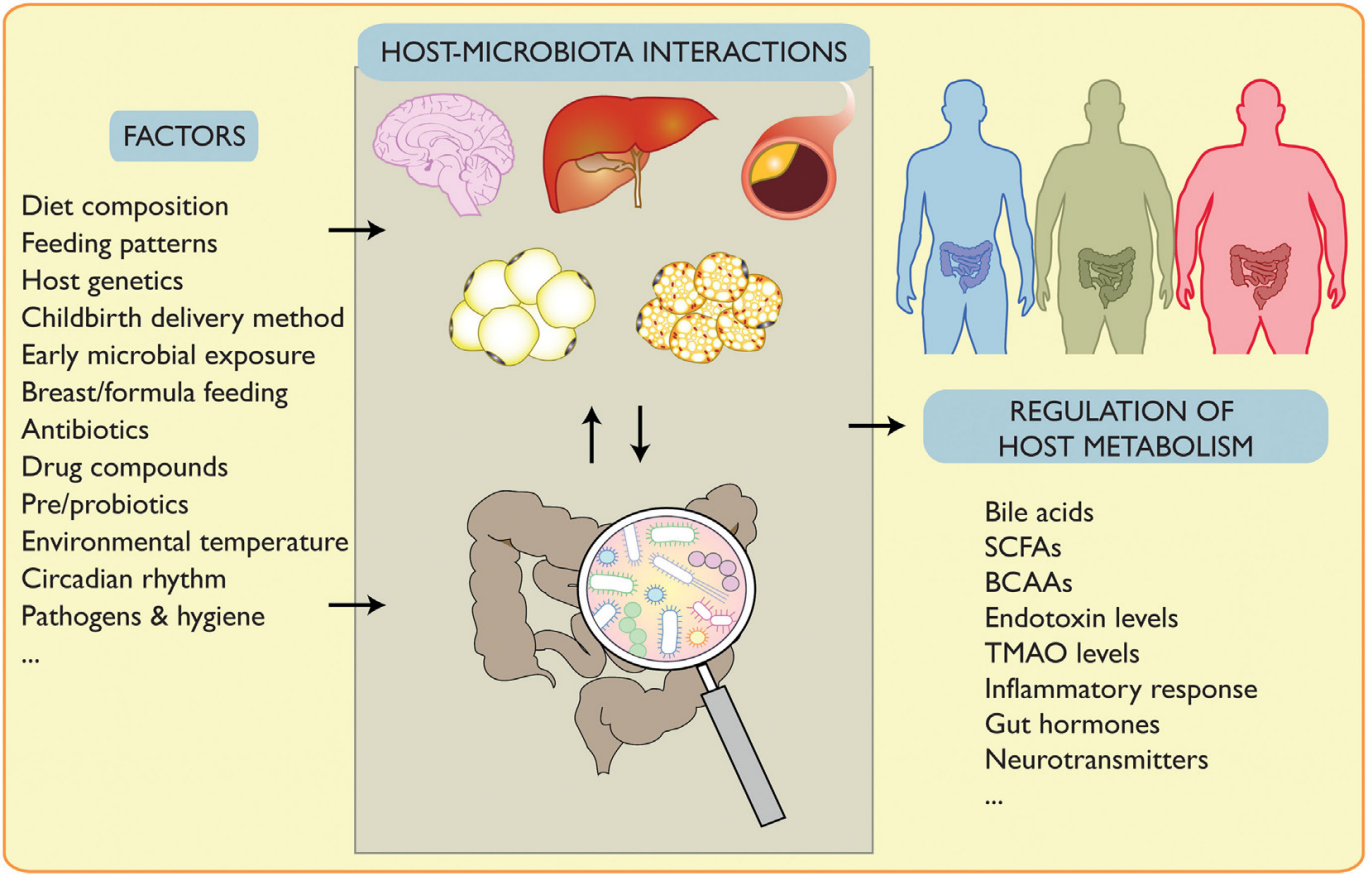
Signals affecting host–microbiota interplay and its regulation of metabolism. Gut microbiota composition is affected by endogenous and exogenous factors such as lifestyle interventions. Changes in the microbiota affect its interplay with several organs and can regulate pathophysiological conditions. This can be mediated by altered bile acids, short chain fatty acids (SCFAs), branched-chain amino acids (BCAAs), endotoxin, trimethylamine *N*-oxide (TMAO), inflammation, gut hormones and neurotransmitters, and potentially other factors.

Using integrative metabolomics–metagenomics approaches, Pedersen and colleagues identified *Prevotella copri* and *Bacteroides vulgatus* as main species positively correlating their branched-chain amino acids (BCAAs) biosynthesis capacity with insulin resistance in humans. When supplementing *P. copri* to mice during HFD, BCAAs circulating levels increased, inducing insulin resistance and glucose intolerance (61). This is in line with several studies showing that elevated BCAAs can lead to metabolic disorders (62, 63) and provide correlation between their levels and diabetic status (64, 65) (Figure 1).

## Dietary Interventions and Therapeutic Potential of Microbiota Reshaping

### Feeding Patterns and Microbiota Compositional Fluctuations

Different lifestyles are associated with changes in microbiota composition, which can result in different efficacy in energy extraction from food and, therefore, impact host metabolism (7, 12). The general microbial composition, as well as the abundance of multiple taxa, undergoes circadian oscillations (66–68). This rhythmicity is dictated by the feeding pattern of the host controlled by its own circadian clock, as genetic depletion of the clock machinery, or its disruption due to jet lag induces dysbiosis and loss of diurnal cycling. In turn, the microbiota too can influence the circadian fluctuation of intestinal epithelial cells (69) and affect intestinal and hepatic metabolism through rhythmic patterns of attachment to the mucosa and metabolomic changes (70). The timing of meals, therefore, influences acute compositional fluctuations in the gut microbiota and modulate microbial-dependent effects on the host. For instance, time-restricted feeding (limiting food access to 10–11 h/day) reduces body weight and improves well-being in overweight individuals (71). While the authors did not explore the subsequent changes in the microbiota of the people involved in their study, in HFD-fed mice, time restriction is associated with a decrease of obesogenic taxa and an increase in beneficial bacteria, thus improving host metabolism (67).

### Dietary Fibers and Prebiotics

Diet composition is one of the most important factors that shape the gut microflora. Diets rich in saturated fatty acids are associated with insulin resistance and adipose inflammation (72), whereas polyunsaturated fats have an insulin-sensitizing role (73). Both kinds of dietary lipids affect metabolism through compositional changes in the gut microbiota and signaling of microbial byproducts to the host (74). Protein intake and protein–carbohydrate ratio also impact the production of multiple bacterial metabolites (75). Carbohydrates constitute an important source of energy for the microbiota and, as mentioned above, their byproducts—the SCFAs influence host metabolism. The reduced fiber intake in western diets is associated with reduced bacterial richness and metabolic disorders (76), both of which can be rescued in overweight individuals by dieting (77, 78). Increased fiber consumption leads to improved postprandial glucose metabolism in response to whole grain-based meals (79) and is associated with an increase in *Prevotella* abundance (80) and a higher ratio of *Prevotella* over *Bacteroides*, the two main genera of the *Bacteroidetes* phylum. During fiber-rich diet, *Prevotella* appears to positively interact with species from the *Actinobacteria, Firmicutes, Proteobacteria*, and *Archaea* phyla to form a niche of bacteria involved in carbohydrate fermentation (81). This contributes to an improved glucose metabolism through increased hepatic glycogen storage (81).

Administration of oligofructose in obese mice regulates appetite, reduces obesity, and the related metabolic disturbances. These improvements are associated with 100-fold increase in the abundance of *Akkermansia muciniphila*, increased growth of *Bifidobacteria*, and *Lactobacilli*, and expression of antimicrobial peptides by the host (82, 83). Studies on healthy and obese individuals demonstrate expansion in *Bifidobacterium* species and *Faecalibacterium prausnitzii* during prebiotic treatment. Prebiotics can also induce satiety by regulating the SCFAs (84) and increasing Peptide YY and GLP1 production by the L cells in the ileon and the colon (85–87). In turn, these enteroendocrine hormones inhibit the hypothalamic orexigenic (hunger-inducing) regions and stimulate the anorexigenic (satiety-inducing) neurons (85–88). Fiber-rich diets also impact the interaction between the microbiota and the intestinal mucosal layer, a barrier that separates the epithelium from direct contact with bacteria, constituting a first level of defense against pathogen infection (89). Prebiotic treatment promotes production of the glucagon-like peptide-2, which increases mucosal barrier function and reduces endotoxin-driven inflammation in obese mice (90). Conversely, disruption or ablation of the mucose layer leads to intestinal inflammation, colitis, and even cancer (91–93). In absence of dietary fibers, the mucus layer is dramatically reduced due to expansion of a mucin-degrading bacterial niche. This causes susceptibility to enteric pathogens (94) and increases the predisposition toward metabolic disorders. Indeed, monocolonization with *Bacteroides thetaiotamicron*, a mucin degrader in absence of other available sources of energy, causes impaired glucose tolerance through decreased hepatic glycogen storage (81). Conversely, fiber-rich diets in humans promote the presence of species from the *Xylanibacter, Prevotella, Butyrivibrio*, and *Treponema* genera, preventing the colonization of intestinal pathogens like *Enterobacteriaceae* (95, 96). A vertical study in mice addressed the long-term effects on microbial changes in response to a low fiber diet. Whereas reverting to a fiber-rich diet within a single generation mostly restores the microbial composition, the loss of microbial taxa under fiber-low diets is not reversible after several generations (97). These results suggest that in addition to the dietary changes, it may be necessary to reintroduce beneficial taxa that are currently lost in the Western microbiota in order to prevent the diseases associated with it.

### Probiotics

Probiotics are live bacteria, usually present in fermented foods, whose intake improves metabolic health. Their supplementation in diet has been associated with protective effects against irritable bowel syndrome (IBS), ulcerative colitis, allergic diseases, and obesity in both rodents and humans. They are mostly Gram-positive bacteria belonging either to the *Lactobacillus* or *Bifidobacterium* genera, although a Gram-negative, non-pathogenic, *Escherichia coli* strain has also a probiotic effect (98). The mechanism of action of probiotics is quite heterogeneous and depends on the specific strain used. The anti-obesity effects include reducing metabolic endotoxemia (99–101), improving endothelial dysfunction in obese mice (102, 103), improving hepatic steatosis (104), and limiting free fatty acids available to the host (105). This wide range of effects is mediated by multiple, mutually linked mechanisms like increased intestinal adhesion and colonization that limit the colonization of less beneficial bacteria, production of metabolites such as SFCA and poly-unsaturated fatty acids (106, 107), release of antibacterial molecules called bacterocins (108), and strengthening of the intestinal epithelial integrity and the intestinal mucus layer (109). Recently, in addition to “traditional” probiotic species, *A. muciniphila* has gained a lot of interest. Abundance of this species is inversely correlated with body weight and insulin resistance, and its increase is another effect of metformin treatment (110). Daily supplementation of *A. muciniphila* in mice ameliorates HFD-induced metabolic dysfunctions (111), and prevents the increased intestinal absorptive surface and caloric uptake during cold exposure (26). Even pasteurized, *A. muciniphila* potently reduces body weight gain and insulin resistance in obese mice, due to an outer membrane protein called Amuc_1100, which activates TLR2 and restores intestinal gut barrier function (112). Since *A. muciniphila* is a strict anaerobic species, the discovery that it can exert its protective function against metabolic disorders after pasteurization makes it a more manageable and therapeutically interesting tool.

### Fecal Transplants

Another way to restore a dysbiotic state and reintroduce beneficial taxa is through fecal microbiota transplant (FMT) from a healthy donor. It is currently mainly used to restore intestinal balance in patients affected by recurrent *Clostridium difficile* infections, with a success rate up to 94% and without adverse effects (113). Since dysbiotic states are clinically similar regardless of the origin, this therapy is currently being tried also for non-infectious intestinal pathologies, like intestinal bowel disease and IBS (114, 115), with first few randomized trials that suggest, at least for IBS, a recovery in bacterial richness after transplantation and an attenuation of the symptoms (114, 116). In the context of the metabolic syndrome, a first human trial on obese Caucasian male subjects showed an increase in peripheral insulin sensitivity in patients receiving allogenic gut microbiota, as well as a tendency to increased hepatic insulin sensitivity (117). Subsequent analyses on this and other cohorts of human patients undergoing FMT (118) have suggested that the stimulation of the recipient microbiota with the donor one has an important impact on the efficiency of the microbial transfer and its persistence in the host and that, therefore, the outcome of FMT depends on the composition of both microbiota (119, 120). With FMT being suggested also for a plethora of other pathologies including anxiety, depression, and even autism (116, 121), increasing our knowledge on the function and the interaction of the gut microflora within itself and with the host will, therefore, be paramount in order to design microbiota-based therapies.

## Perspectives

Dissecting how bacterial cues are sensed and act on host physiology is essential to either modulate the microbiota or mimic its signals in a therapeutic perspective. Nevertheless, the known variability of microbial ecosystems in humans is currently a constraint for standard treatments. We can envision an approach where the advances in gut microbiota profiling applied to personalized medicine could allow the definition of pipelines for treatments aiming at re-establishing a healthy microflora. These considerations can potentially overcome the current obstacles in single taxa reintroduction or fecal microbiota transfer and could rely on sequential treatments to reopen ecological niches for beneficial bacteria.

## Author Contributions

The authors reviewed literature, conceived, and wrote the manuscript and artwork.

## Conflict of Interest Statement

The authors declare that the research was conducted in the absence of any commercial or financial relationships that could be construed as a potential conflict of interest.
